# Acquired Deficiency of A20 Results in Rapid Apoptosis, Systemic Inflammation, and Abnormal Hematopoietic Stem Cell Function

**DOI:** 10.1371/journal.pone.0087425

**Published:** 2014-01-31

**Authors:** Akiko Nagamachi, Yuichiro Nakata, Takeshi Ueda, Norimasa Yamasaki, Yasuhiro Ebihara, Kohichiro Tsuji, Zen-ichiro Honda, Keiyo Takubo, Toshio Suda, Hideaki Oda, Toshiya Inaba, Hiroaki Honda

**Affiliations:** 1 Department of Molecular Oncology, Research Institute of Radiation Biology and Medicine, Hiroshima University, Minami-ku, Hiroshima, Japan; 2 Department of Disease Model, Research Institute of Radiation Biology and Medicine, Hiroshima University, Minami-ku, Hiroshima, Japan; 3 Division of Cellular Therapy, Advanced Clinical Research Center, The Institute of Medical Science, The University of Tokyo, Minato-ku, Tokyo, Japan; 4 Health Care Center and Graduate School of Humanities and Sciences, Institute of Environmental Science for Human Life, Ochanomizu University, Bunkyo-ku, Tokyo, Japan; 5 Department of Cell Differentiation, The Sakaguchi Laboratory of Developmental Biology, Keio University School of Medicine, Shinjuku-ku, Tokyo, Japan; 6 Department of Pathology, Tokyo Women’s Medical University, Shinjuku-ku, Tokyo, Japan; Emory University, United States of America

## Abstract

A20 is a negative regulator of NF-κB, and mutational loss of A20 expression is involved in the pathogenesis of autoimmune diseases and B-cell lymphomas. To clarify the role of A20 in adult hematopoiesis, we generated conditional *A20* knockout mice (*A20^flox/flox^*) and crossed them with *Mx*–*1Cre* (*MxCre* ^+^) and *ERT2Cre* (*ERT2Cre* ^+^) transgenic mice in which Cre is inducibly activated by endogenous interferon and exogenous tamoxifen, respectively. *A20^flox/flox^ MxCre* ^+^ (*A20Mx*) mice spontaneously exhibited myeloid proliferation, B cell apoptosis, and anemia with overproduction of pro-inflammatory cytokines. Bone marrow transplantation demonstrated that these changes were caused by hematopoietic cells. NF-κB was constitutively activated in *A20Mx* hematopoietic stem cells (HSCs), which caused enhanced cell cycle entry and impaired repopulating ability. Tamoxifen stimulation of *A20^flox/flox^ ERT2Cre* ^+^ (*A20ERT2*) mice induced fulminant apoptosis and subsequent myeloproliferation, lymphocytopenia, and progressive anemia with excessive production of pro-inflammatory cytokines, as observed in *A20Mx* mice. These results demonstrate that A20 plays essential roles in the homeostasis of adult hematopoiesis by preventing apoptosis and inflammation. Our findings provide insights into the mechanism underlying A20 dysfunction and human diseases in which A20 expression is impaired.

## Introduction

NF-κB plays fundamental roles in various physiological and pathological processes, such as immunity, apoptosis, inflammation, and cancer [1], [2], [3]. In an unstimulated state, NF-κB is sequestered in the cytoplasm by binding to IκB proteins. Upon activation by external stimuli, IκB proteins are phosphorylated by the IκB kinase (IKK) complex and then degraded by ubiquitination. NF-κB is released and translocates to the nucleus where it drives the expression of target genes [1], [2], [3].

A20, also known as tumor necrosis factor alpha-induced protein 3 (TNFAIP3), now emerges as a major negative regulator of NF-κB signaling [4], [5]. A20 comprises an ovarian tumor (OTU) domain at its N-terminus and seven Zn-finger motifs. The OTU domain is predicted to have deubiquitinating protease activity, and the Zn finger motifs possess E3 ubiquitin ligase and ubiquitin-binding activities [4], [5]. Thus, A20, acting as a ubiquitin-modifying protein, may participate in a negative feedback loop controlling NF-κB signaling [4], [5]. The most compelling evidence that A20 plays an essential role in inhibiting inflammation are results of a gene knockout experiment in which A20 deficient mice prematurely died because of severe systemic inflammation and cachexia [6].

A20 is involved in various human diseases, including hematopoietic malignancies. Frequent loss of A20 expression in B-cell lymphomas caused by biallelic deletions and/or point mutations [7], [8] indicates that A20 functions as a tumor suppressor in the hematopoietic system. Moreover, single nucleotide polymorphisms in *A20* are associated with autoimmune and inflammatory diseases, such as systemic lupus erythematosus (SLE) [9], [10], [11], rheumatoid arthritis (RA) [12], [13], and Crohn’s disease [14].

An approach to determine whether there is a causative association between A20 mutations and pathogenesis employs mice to target A20 in a tissue-specific manner. A number of A20 conditional knockout (cKO) mice have been generated for this purpose. For example, B cell-specific deletion of *A20* using a *CD19*–*Cre* transgene results in hyper-responsiveness of B cells and causes autoimmune disease similar to SLE [15], [16], [17]. Deletion of A20 from dendritic or myeloid cells using *CD11c–Cre* or *LysM–Cre* transgenes, respectively, also induced autoimmune disease. The former exhibited an SLE-like phenotype [18], and the latter developed an RA-like disease [19]. Moreover, *Villin–Cre* transgenic mice harboring a deletion of A20 from their epithelial intestinal cells showed susceptibility to dextran sodium sulfate-induced colitis [20].

Although these studies provide important insights into the role of A20 as a suppressor of tumorigenesis and autoimmunity, its role(s) in the normal functioning of the hematopoietic system of adults remains to be determined. To address this issue, we created mice in which A20 expression can be inducibly and preferentially ablated in hematopoietic cells.

## Materials and Methods

### Mice

The detailed procedures for constructing the targeting vector and generating the *A20^flox/flox^* mice are described in Text S1 (*A20* cKO mice have been deposited in RIKEN BioResource Center (http://www.brc.riken.jp/inf/en/index.shtml, RBRC05494). *A20^flox/flox^* mice were crossed with *Mx*–*1Cre* (*MxCre* ^+^) transgenic mice [21] and *ERT2Cre* (*ERT2Cre* ^+^) transgenic mice (C57BL/6-Gt(ROSA)26Sor*^tm1(cre/Est1)Arte^*, purchased from Taconic) to generate *A20^flox/flox^ MxCre* ^+^ and *A20^flox/flox^ ERT2Cre* ^+^ mice, respectively. Mice backcrossed with the C57BL/6-Ly5.2 background at least seven times were used here. This study was carried out in strict accordance with the recommendations in the Guide for the Care and Use of Laboratory Animals of the Hiroshima University Animal Research Committee. The protocol was approved by the Committee on the Ethics of Animal Experiments of the Hiroshima University (Permit Number: A13-13). All mice were maintained according to the guidelines of the Institute of Laboratory Animal Science of Hiroshima University, all surgeries were performed under sodium pentobarbital anesthesia and all efforts were made to minimize suffering.

### Western Blotting, Flow Cytometry, and Histopathology

Western blotting, flow cytometry, and histopathology were performed as previously described [22], [23], [24]. Antibodies and a staining kit used in these analyses are listed in Table S1.

### Measurement of Serum Cytokine Concentration

Concentrations of pro-inflammatory cytokines (TNF-α, IFN-γ, GM-CSF, IL-1β, and IL-6) were measured using a BD Cytometric Bead Array Flex Set Kit (BD Biosciences, San Diego, CA, USA) according to the manufacturer’s instructions.

### Colony Formation Assay

The colony formation assay was performed as previously described [25].

### Bone Marrow Transplantation (BMT), Competitive Repopulation, and Cell Cycle Analyses

Transplantation of bone marrow (BM) cells, competitive repopulation, and short-term BrdU incorporation assays were performed as previously described [24].

## Results

### *A20^ flox^* ^/*flox*^ *MxCre* ^+^ Mice Exhibited Severe Inflammation, B Lymphocyte Apoptosis, and Premature Death

To conditionally ablate A20 function, we generated mice in which exon 3 of *A20* was flanked by two *loxP* sites (A20*^flox/flox^* mice, Fig. S1A and S1B). To examine the role of A20 in hematopoietic homeostasis, we crossed A20*^flox/flox^* mice with *Mx*–*1Cre* transgenic (*MxCre* ^+^) mice in which *Cre* is placed under the control of IFN-responsive *Mx*–*1* promoter [21]. Lack of A20 expression in A20*^flox/flox^ MxCre* ^+^ mice was confirmed by western blotting of spleen extracts prepared from A20*^flox/flox^ MxCre* ^−^ and A20*^flox/flox^ MxCre* ^+^ mice (hereafter referred to as *control* and *A20Mx* mice, respectively) using an anti-A20 antibody (left panel of Fig. S1C).

**Figure 1:**
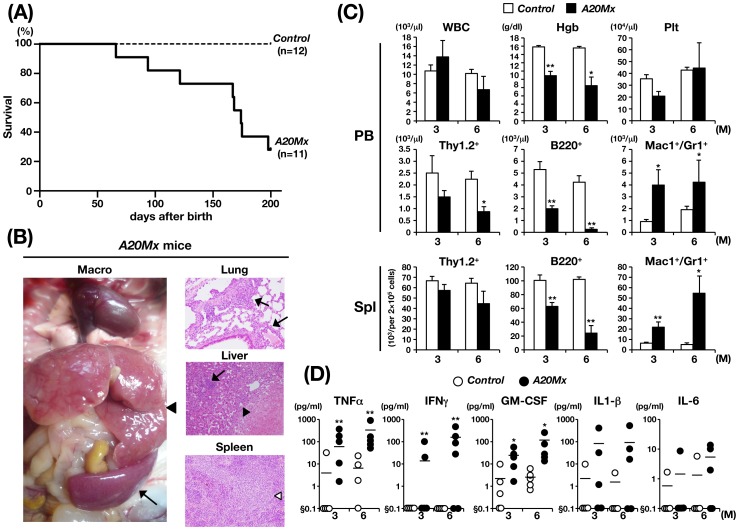
Analysis of *A20Mx* mice. (A) Kaplan–Meier survival curves. (B) Macroscopic appearance and pathological images of an *A20Mx* mouse six months after birth. In the left panel, the enlarged liver and spleen are indicated by an arrowhead and an arrow, respectively. In the right panel, infiltrated myeloid cells in the lung and liver, granuloma formation in the liver, and destruction of spleen architecture are indicated by arrows, an arrowhead, and a white arrowhead, respectively. (C) Time-dependent changes in hematopoietic parameters in the PB and spleen. *p<0.05 and **p<0.01 (Student’s *t*-test). (D) Serum concentrations of pro-inflammatory cytokines. *p<0.05 and **p<0.01 (Student’s *t*-test). § below standard range and out of invertable range.

Although *A20Mx* mice were apparently normal at birth, they exhibited spontaneous emaciation and cachexia without stimulation by polyinosinic:polycytidylic acid (pIpC), which is a strong and transient inducer of IFN, and most mice died within six months after birth (Fig. 1A). Hematological analysis of moribund mice revealed anemia, proliferation of myeloid cells, and reduction of B lymphoid cells in the peripheral blood (PB) (Table S2). The macroscopic appearance of the mice was uniformly characterized by massive hepatomegaly and enlarged spleens (indicated by an arrowhead and an arrow, respectively, in the left panel of Fig. 1B), which were frequently associated with lymph node (LN) swelling (Table S2). Pathological analysis revealed infiltration of the lung and liver by hematopoietic cells (indicated by arrows in the right top and middle panels of Fig. 1B), formation of granulomas in the liver (indicated by an arrowhead in the right middle panel of Fig. 1B), and destruction of spleen architecture caused by the proliferation of white blood cells (indicated by a white arrowhead in the right bottom panel of Fig. 1B). Higher magnification of the spleen showed that the most of the proliferated cells were with segmented or multi-lobulated nuclei, strongly suggesting that these cells were of myeloid in origin (indicated by arrowheads in the left panel of Fig. S2).

We then analyzed the time-dependent changes in hematopoietic parameters in *control* and *A20Mx* mice. The white blood cell count (WBC), hemoglobin concentration (Hgb), and platelet (Plt) number in the PB, and the absolute numbers of B lymphoid (B220^+^), T lymphoid (Thy1.2^+^), and myeloid (Mac1^+^Gr1^+^) cells in the PB and spleen were analyzed at three and six months after birth. Although no significant difference was observed in the WBC count and Plt number between the two groups, *A20Mx* mice exhibited progressive anemia (top panels of Fig. 1C). In addition, lineage analysis of WBCs in the PB and spleen revealed that the numbers of myeloid cells were significantly increased, whereas those of B lymphoid cells were significantly decreased in *A20Mx* mice compared with *control* mice (middle and bottom panels of Fig. 1C). To clarify the mechanism of B-cell reduction in *A20Mx* mice, spleens of *A20Mx* mice and control littermates were subjected to and anti-B-cell and TUNEL double staining. As shown in Fig. S3, a significant portion of B lymphocytes in the *A20Mx* spleen was positive for TUNEL, indicating that the B cell reduction in *A20Mx* mice was due to apoptosis.

Because A20 inhibits NF-κB signaling and suppresses inflammatory pathway activity [4], [5], we reasoned that the aforementioned findings were caused by sustained inflammatory responses. Therefore, serum concentrations of pro-inflammatory cytokines, including TNF-α, IFN-γ, GM-CSF, IL-1β, and IL-6, were measured and compared between the two groups. The concentrations of these cytokines were higher in *A20Mx* mice than in *control* mice, and those of TNF-α, IFN-γ, and GM-CSF were significantly increased (Fig. 1D). These results indicate that cytokine-mediated inflammation induced the proliferation of myeloid cells and other hematopoietic abnormalities, which subsequently damaged internal organs and eventually caused premature death of *A20Mx* mice.

### Transfer of the Aberrant *A20Mx* Hematopoietic Phenotype to Naïve Mice

**Figure 2:**
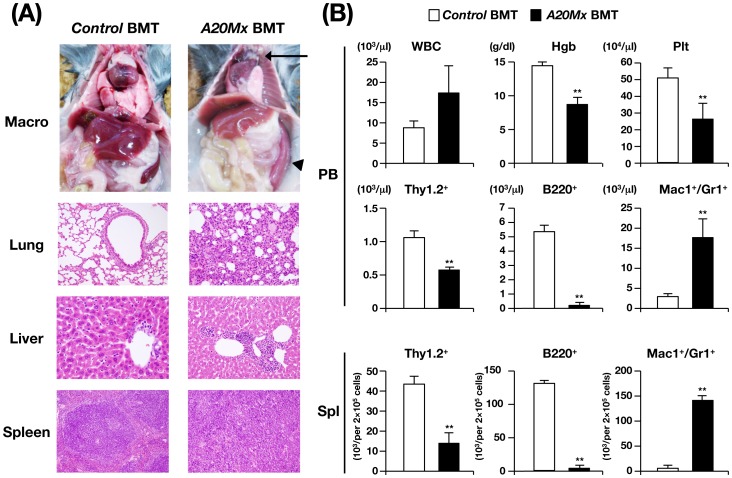
Analysis of *A20Mx* BMT mice. (A) Macroscopic appearance and pathological images of *control* BMT and *A20Mx* BMT mice. The atrophic thymus and enlarged spleen are indicated by an arrow and an arrowhead, respectively. Note the infiltrating hematopoietic cells in the lung and liver and the destruction of spleen architecture caused by excessive proliferation of myeloid cells in *A20Mx* BMT mice. (B) Time-dependent changes in hematopoietic parameters in the PB and spleen. **p<0.01 (Student’s *t*-test).

To determine the cellular origin of the severe inflammation observed in *A20Mx* mice, we performed BMT assays. Mononuclear BM cells derived from *control* and *A20Mx* mice were transplanted into lethally irradiated syngeneic recipient mice (hereafter, mice transplanted with *control* and *A20Mx* BM cells are referred to as *control* and *A20Mx* BMT mice, respectively). One month after BMT, all *A20Mx* BMT mice became moribund and were sacrificed to assess hematopoietic and pathological changes. Macroscopically, *A20Mx* BMT mice exhibited thymic atrophy and splenic enlargement (indicated by an arrow and an arrowhead, respectively, in the right top panel of Fig. 2A). Pathological analysis revealed marked infiltration of hematopoietic cells in the lung and liver (right 2^nd^ and 3^rd^ panels of Fig. 2A) and massive proliferation of the same cell types in the spleen (right bottom panel of Fig. 2A) of *A20* BMT mice. The proliferated cells in the spleen were with segmented or multi-lobulated nuclei, indicating that the cells were mature granulocytes, as observed in the *A20Mx* spleen (indicated by arrowheads in the right panel of Fig. S2). Hematopoietic analysis showed an elevated WBC count, reduced Hgb concentration, and decreased Plt number in *A20Mx* BMT mice (top panel of Fig. 2B). Lineage analysis of the PB and spleen revealed a significant increase in the numbers of myeloid cells and a significant decrease in the numbers of B and T lymphoid cells in both tissues (middle and bottom panels of Fig. 2B). The abnormal phenotypes of *A20Mx* BMT mice were similar to those of *A20Mx* mice, indicating that the inflammatory changes detected in *A20Mx* mice were caused by hematopoietic cells.

### Impaired Repopulation Ability, Enhanced Cell Cycle Entry, and Constitutive NF-κB Activation in Hematopoietic Stem Cells of *A20Mx* Mice

We then investigated whether the abnormal phenotypes of *A20Mx* and *A20Mx* BMT mice were induced by hematopoietic stem cells (HSCs). HSCs are usually defined as “lineage marker (Lin)^ −^, Sca-1^+^ and c-Kit^+^ (LSK)” cells. However, in *A20Mx* mice, this method is not appropriate, since serum concentration of IFN-γ is elevated and the expression level of Sca-1 was reported to be up-regulated by IFNs [26]. Thus, to isolate HSCs, we used other markers, “Lin^−^, CD48^−^, CD150^+^”, called as SLAM-code, which distinguishes HSCs from progenitor cells [27].

**Figure 3:**
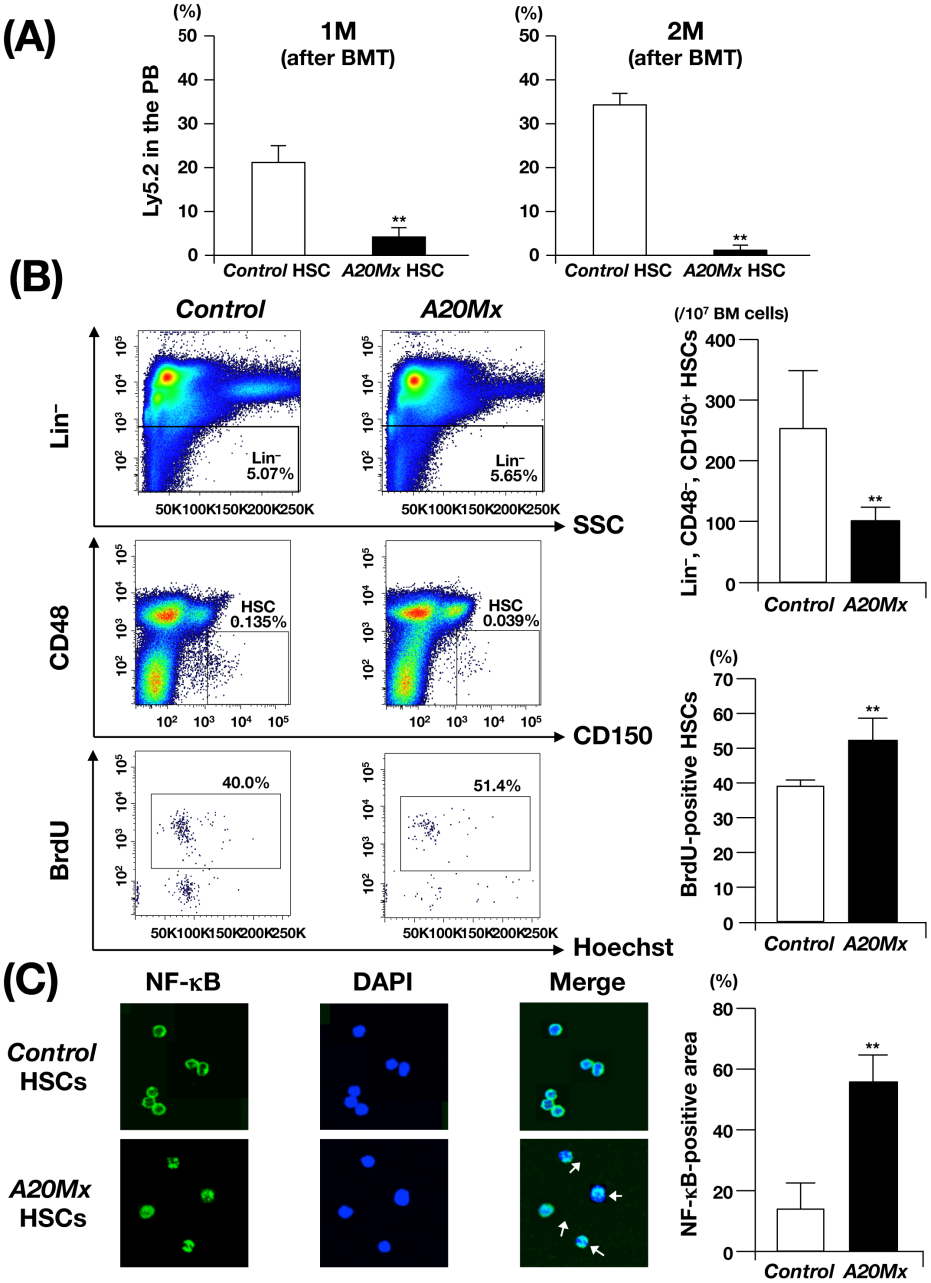
Analysis of HSCs in *control* and *A20Mx* mice. (A) The percentages of Ly5.2^+^ cells in the PB of *control* and *A20Mx* BMT mice at 1 and 2 months after BMT are shown. **p<0.01 (Student’s *t*-test). (B) Cell number and BrdU incorporation in *control* and *A20Mx* HSCs. Representative results of flow cytometry is shown in the left panels and the HSC number and percentage of BrdU-positive cells are shown in the right column. **p<0.01 (Student’s *t*-test). (C) Intracellular localization of NF-κB in *control* and *A20Mx* HSCs. Immunofluorescence staining of NF-κB and percentages of the NF-κB-positive areas in the cells are shown in the left panels and right column, respectively. Cells in which NF-κB is translocated into the nucleus are indicated by arrows. **p<0.01 (Student’s *t*-test).

HSCs isolated from *control* and *A20Mx* mice (Ly5.2^+^) were transplanted into lethally irradiated Ly5.1^+^ recipients together with Ly5.1^+^ BM MNCs as competitors (hereafter the recipient mice transplanted with *control* and *A20Mx* HSCs are referred to as *control* and *A20Mx* HSC BMT mice, respectively). Interestingly, in contrast that the percentage of Ly5.2^+^ cells in the PB of *control* HSC BMT mice increased after transplantation and accounted for approximately 40% of WBCs at two months, that of *A20Mx* HSCs BMT mice was significantly less and did not reach 10% of WBCs during the observation period (Fig. 3A). These results indicate severely impaired repopulating ability of HSCs in *A20Mx* mice and suggest abnormal proliferation/differentiation status and cell cycle kinetics of *A20Mx* HSCs.

To address this issue, HSCs isolated from *control* and *A20Mx* mice were subjected to cell counting and short-term 5-bromo-2′-deoxyuridine (BrdU) uptake assay. As shown in Fig. 3B, Although the absolute number of HSCs was considerably decreased in *A20Mx* mice as compared to *control* mice, BrdU uptake by *A20Mx* HSCs was significantly higher than that of *control* HSCs, indicating that *A20Mx* HSCs entered the cell cycle at a higher rate.

A20 is a negative regulator of NF-κB [4], [5] and NF-κB activation is detected by its translocation from the cytoplasm to the nucleus [1], [28]. To investigate whether NF-κB activation underlies the abnormal behaviors of HSCs, the intracellular localization of NF-κB in HSCs of *control* and *A20Mx* mice were examined by immunofluorescently staining with an anti-NF-κB antibody. In contrast that NF-κB signals in *control* HSCs were mainly observed in the cytoplasm, those in *A20Mx* HSCs were significantly detected in the nucleus (Fig. 3C). These results indicate that NF-κB was constitutively activated in *A20Mx* HSCs, which caused increased cell cycle entry, perturbed their stemness and eventually impaired the repopulating activity.

### Rapid Apoptosis and Subsequent Anemia, Myeloid Proliferation, and Reduced Numbers of Lymphocytes Induced by Acquired A20 Deficiency in Hematopoietic Cells

Because the expression of Cre in *MxCre* ^+^ mice was controlled by the IFN-responsive element [21], the activity of Cre was very likely influenced by endogenous IFN. Therefore, we used an *ERT2Cre* transgenic (*ERT2Cre* ^+^) system in which Cre is fused to a mutated estrogen receptor and can only be activated by exogenously administered tamoxifen. For this purpose, we crossed *A20^flox^* ^/*flox*^ mice with *ERT2Cre* ^+^ mice and generated *A20^flox^* ^/*flox*^ *ERT2Cre* ^+^ mice (hereafter referred to as *A20ERT2* mice). *A20^flox^* ^/*flox*^ *ERT2Cre* ^−^ littermates were used as controls. *A20ERT2* mice did not spontaneously develop abnormal phenotypes in contrast to *A20Mx* mice. However, after tamoxifen administration, *A20ERT2* mice became rapidly moribund, exhibited a marked decrease in the number of hematopoietic cells and died within several days (not shown). Macroscopical examinations revealed that the thymus was atrophic, the spleen was pale, and liver had white spots (not shown). Pathological analysis revealed that massive apoptosis occurred in major organs, including hematopoietic tissues such as the thymus, spleen, liver, and BM (Fig. S4A). Microemboli and necrotic areas were also observed in the liver (indicated by an arrow and arrowheads in Fig. S4A), suggesting that the white spots in the liver were caused by ischemic necrosis. The measurement of pro-inflammatory cytokines revealed that the concentrations of these cytokines were higher in *A20ERT2* mice than in *control* mice, and those of TNF-α, IFN-γ, and IL-6 were significantly increased (Fig. S4B). These results indicate that loss of A20 in adult mice induced fulminant apoptosis, possibly via rapid elevation of pro-inflammatory cytokines.

**Figure 4:**
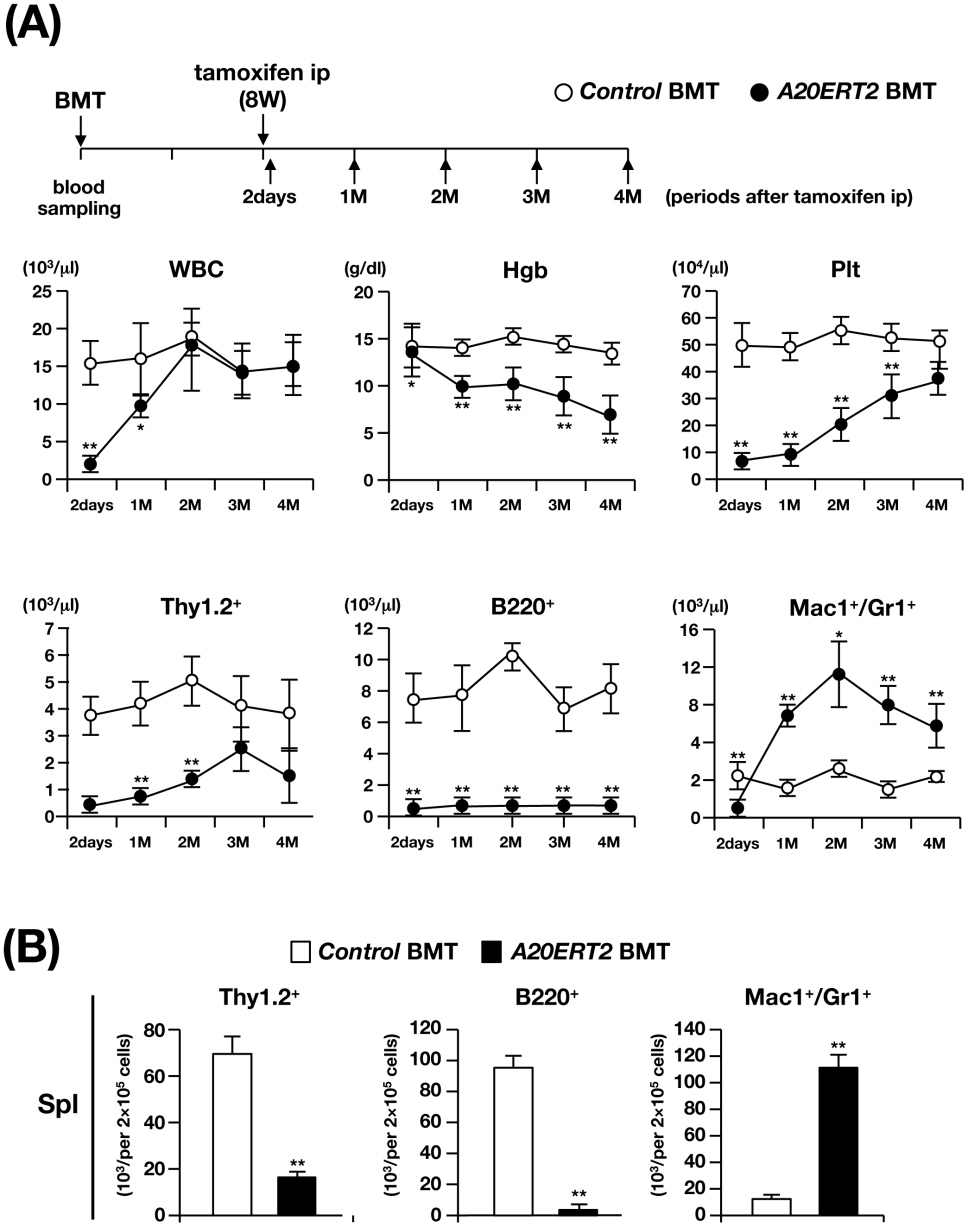
Analysis of *control* and *A20ERT2* BMT mice. (A) Time schedule of BMT, tamoxifen ip, blood sampling, and time-dependent changes in hematopoietic parameters in the PB. **p<0.01 (Student’s *t*-test). (B) Percentages of Thy1.2^+^, B220^+^, and Mac1^+^/Gr1^+^ cells in the spleen 4 M after transplantation. **p<0.01 (Student’s *t*-test).

We next investigated the effect of deleting *A20* from hematopoietic cells by transplanting *control* and *A20ERT2* BM cells into lethally irradiated syngeneic mice and then administrating tamoxifen (mice transplanted with *A20ERT2* BM cells are hereafter referred to as *A20ERT2* BMT mice). Before tamoxifen stimulation, the number of transplanted Ly5.2^+^ cells was comparably increased in control BMT and *A20ERT2* BMT mice, and there was no significant difference in the values of hematopoietic parameters between them (not shown). Tamoxifen was administered eight weeks after transplantation [29] (Fig. 4A), and two days later, all *A20ERT2* BMT mice became moribund with marked decrease in WBC count and Plt number in the PB (“2 days” at top panels of Fig. 4A).

Tamoxifen-treated *A20ERT2* BMT mice survived this stage, in contrast to *A20ERT2* mice that died after several days after tamoxifen administration, probably because there were no severe apoptotic changes in the non-hematopoietic tissues. Examination of PB parameters showed that *A20ERT2* BMT mice exhibited progressive anemia, proliferation of myeloid cells, and reduction in lymphocyte number, particularly that of B cells, as observed in *A20Mx* and *A20Mx* BMT mice (bottom panels of Fig. 4A). The analysis of the spleen four months after tamoxifen stimulation showed a marked reduction of B and T lymphocytes and a significant increase of myeloid cells (Fig. 4B).

**Figure 5:**
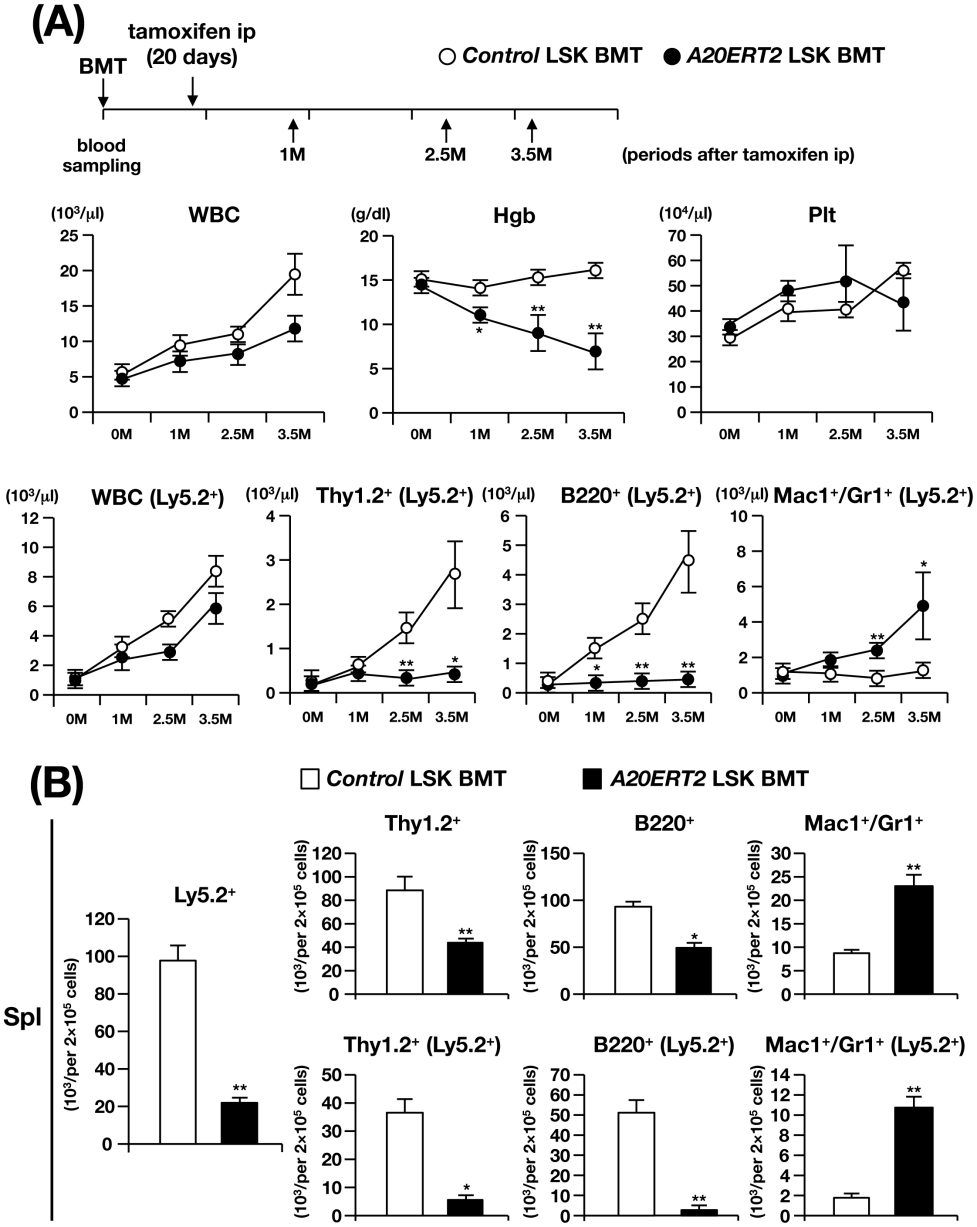
Analysis of *control* and *A20ERT2* LSK BMT mice. (A) Time schedule of BMT, tamoxifen ip, blood sampling, and time-dependent changes of hematopoietic parameters and percentages of Ly5.2^+^ cells in the PB. *p<0.05 and **p<0.01 (Student’s *t*-test). (B) Percentages of different hematopoietic compartments and the population of Ly5.2^+^ cells in the spleen 3.5 M after transplantation. **p<0.01 (Student’s *t*-test).

To further investigate whether the changes in the hematopoietic cell population in tamoxifen-treated *A20ERT2* BMT mice were caused by HSCs, competitive repopulation assays were performed. Unlike to the MxCre system, the ERT2Cre system does not respond to endogenous IFNs and no hematological abnormalities were found in the unstimulated state, we used LKS markers to isolate HSCs. LSK cells isolated from control and *A20ERT2* mice were transplanted to the recipient mice and tamoxifen was administered 20 days after transplantation [30]. The recipient mice transplanted with *A20ERT2* LSK cells (hereafter referred to as *A20ERT2* LSK BMT mice, Fig. 5A). While *control* LSK BMT mice were healthy during the observation period, *A20ERT2* LSK BMT mice became gradually emaciated and were sacrificed at 3.5 months after transplantation. The mice showed thymic atrophy and splenomegaly (not shown), and hematopoietic analysis demonstrated anemia and decreased numbers of Ly5.2^+^ lymphocytes in the PB, elevation of myeloid cell number, and reduction of lymphoid cell numbers in the total and Ly5.2^+^ fractions of the spleen (Fig. 5A and 5B). These abnormalities were similar to those observed in *A20ERT2* BMT as well as *A20Mx* and *A20Mx* BMT mice. Therefore, we conclude that the abnormalities observed in *A20ERT2* LSK BMT mice can be primarily attributed to the HSCs. Moreover, these abnormalities were found not only in the donor (Ly5.2^+^) cells but also in the competitor cells (Ly5.1^+^), indicating that the changes were propagated by cell–cell contact and/or humoral factors, such as pro-inflammatory cytokines.

## Discussion

A20 plays an essential role in inflammation, immune system, apoptosis, and tumor suppression [4], [5]. The biological roles of A20 in development and in individual tissues have been analyzed using *A20* KO mice [6] and *A20* cKO mice crossed with various Cre transgenic mice [15], [16], [17], [18], [19], [20], [31], respectively. However, to the best of our knowledge, the effect of acquired A20 deficiency on the adult hematopoietic system has not been reported. To address this issue, we generated *A20* cKO mice and crossed them with two different inducible Cre transgenic mice; *Mx1–Cre* and *ERT2Cre* transgenic mice in which Cre is activated in response to endogenous IFN or exogenous tamoxifen, respectively.

A20*^flox/flox^ MxCre* ^+^ (*A20Mx*) mice gradually debilitated and died several months after birth (Fig. 1A) and hematopoietic, pathological, and serological analyses revealed massive inflammatory changes in these mice (Fig. 1B–D). These images of the lung and liver (Fig. 1B), strongly suggest that the mice died of respiratory failure and/or liver dysfunction due to excessive infiltration of hematopoietic cells. Because the *Mx*–*1* promoter is an IFN-responsive element, it is likely that cells that responded to endogenous IFNs, such as macrophages and lymphocytes, lost the A20 gene, did not attenuate IFN-mediated signaling, and continuously produced pro-inflammatory cytokines, which eventually caused severe systemic inflammation and premature death.

To determine whether the phenotypes of *A20Mx* mice could be primarily attributed to hematopoietic cells, BMT assays were performed. Mice transplanted with *A20Mx* BM cells exhibited abnormal phenotypes similar to those of *A20Mx* mice, indicating that these changes were induced by hematopoietic cells (Fig. 2). We also determined the ability of hematopoietic cells of *control* and *A20Mx* mice to form colonies (Fig. S5). When cultured *in vitro* with SCF+IL3+Epo or SCF+IL7, hematopoietic cells from *control* and *A20Mx* mice formed similar numbers of colonies with similar morphology. Thus, the hematopoietic abnormalities observed in *A20Mx* mice were mainly attributed to excessive production of pro-inflammatory cytokines and systemic inflammation.

To further investigate the effect of A20 deficiency on HSC activity, we performed competitive repopulation assays in which Lin^−^, CD48^−^, CD150^+^ HSCs cells in control or *A20Mx* mice (Ly5.2) were transplanted into recipients with Ly5.1 competitors. Interestingly, the repopulation ability of *A20Mx* HSCs was significantly impaired as compared to that of *control* HSCs (Fig. 3A). In addition, BrdU incorporation assays showed an enhanced proliferative ability of *A20Mx* HSCs (Fig. 3B). Furthermore, NF-κB was activated in a substantial portion of *A20Mx* LT-HSCs as revealed by its presence in the nucleus (Fig. 3C). These results indicated that the loss of A20 in the hematopoietic system activated HSCs, entered them into the cell cycle, and eventually impaired the repopulation ability. Quiescence is an important characteristic for HSCs to maintain their stemness and engraftment activity. Our findings in this regard are consistent with those of previous studies demonstrating that cycling HSCs lose its repopulating ability [32], [33], [34]. Whether HSCs sense and directly respond to inflammation is controversial [35], [36]. Our results suggest that HSCs could be a direct target of systemic inflammation and support the idea that TNF-α and IFN-γ are major regulators of inflammatory responses of HSCs, as documented in previous studies [34], [37].

Next, we used *ERT2Cre* mice that respond to exogenously administered tamoxifen but not to endogenous estrogens. As expected, unlike *A20Mx* mice, *A20ERT2* mice did not exhibit an abnormal phenotype before tamoxifen stimulation. However, tamoxifen administration induced rapid death of the *A20ERT2* mice, which we attribute to severe apoptosis (Fig. S4). Moreover, there was a marked decrease in the numbers of hematopoietic cells in *A20ERT2* BMT mice following tamoxifen treatment (Fig. 4). These results indicate that loss of A20 induces rapid systemic apoptosis of hematopoietic cells.

The roles of A20 in apoptosis differ among published studies. For example, certain A20 deficient cells such as fibroblasts, splenocytes, and enterocytes exhibit enhanced sensitivity to TNF-α-mediated apoptosis [6], [20]. In contrast, re-expressing A20 in A20 deficient lymphoma cells promotes apoptosis [7], [8]. These findings indicate that A20 might play reciprocal roles in cell survival and cell death, depending on the cell types and cell contexts. Accordingly, our present results demonstrate that acquired A20 deficiency induces fulminant apoptosis in all cell types, including hematopoietic cells. This suggests that A20 primarily prevents apoptosis in adult tissues. There is evidence that the interaction of A20 with caspase-8 might play a role in this process [38].

The features shared by *A20ERT2* and *A20ERT2* LSK BMT included an increase in the number of myeloid cells, a decrease in the number of lymphocytes, and anemia, and similar to those of *A20Mx* and *A20Mx* BMT mice (Figs. 1, 2, 4, and 5). Thus, cytokine synthesis by hematopoietic cells that survived the apoptotic stage was uncontrolled and caused fulminant inflammation. Proliferation of myeloid cells would be a direct effect of increased production of pro-inflammatory cytokines, and anemia would be attributed to inflammation-induced impaired erythropoiesis as previously described [39]. In contrast, the mechanism responsible for reducing lymphocyte numbers, particularly those of B cells, remains to be clarified. Pathological analysis revealed that B lymphocytes underwent apoptosis in the spleen (Fig. S3), in contrast to previous reports, which show that in B cell-specific A20 deficient mice, B lymphocytes were hyper-reactive to exogenous stimuli and resistant to apoptosis [15], [16], [17]. The mechanism(s) underlying this discrepancy is unknown. One possibility is that B cell behaviors in response to A20 deficiency might differ, depending on the developmental stages at which A20 is deleted (namely, in B lineage-committed cells or in more primitive cells including HSCs). Another possibility is that death factor(s) secreted by systemic inflammation, such as TNF-α might affect lymphocytes and induce apoptosis.

The role of A20 in B cell development/differentiation is of particular interest, because hematopoietic malignancies with A20 mutations are exclusively B lineage lymphomas [7], [8]. Mice with deletions of *A20* in the B lineage cells exhibit autoimmune-like diseases but fail to develop lymphoproliferative disorders [15], [16], [17]. Moreover, MALT lymphoma, a subtype of B-cell lymphoma in which A20 is frequently mutated, originates from mutated hematopoietic stem–early progenitor cells [40]. Therefore, A20 function must be abrogated in early progenitor cells to mimic B-cell lymphomas harboring *A20* mutations, as accomplished here. However, because apoptosis decreased the number of B cells in *A20Mx* and *A20ERT2* mice (Figs. S3 and S4), anti-apoptotic signal(s) might be necessary to exhibit a fully malignant phenotype. To address this possibility, we are crossing *A20Mx* mice with *p53* knockout mice to prevent apoptosis, and with *CagA* transgenic mice, a model for *Helicobactor pylori* infection that plays a pivotal role in the initiation of MALT lymphoma [41].

In summary, we generated mice in which *A20* can be inducibly and preferentially deleted from hematopoietic cells and found that acquired loss of A20 induced fulminant apoptosis and subsequent systemic inflammation. Our results demonstrate the essential role of A20 in the maintenance of adult hematopoietic cell homeostasis and provide insights into the mechanism responsible for A20 dysfunction and human diseases with *A20* mutations.

## Supporting Information

Figure S1 **Generation of** ***A20*** **conditional knockout mice.** (A) Targeting strategy. Exon 3 of mouse *A20* was *floxed* and the *Frt*-flanked *Neo*-resistance gene was removed using Flp recombinase. The positions of a 5′ probe for Southern blotting and P1 and P2 primers for 3′ genomic PCR analysis are shown. Restriction sites: *Ba*, *BamH*I; *Sac*, *Sac*I; *Stu*, *Stu*I; *Sal*, *Sal*I; *Bg*, *Bgl*II. (B) Southern blotting and genomic PCR using a 5′ probe and 3′ primers, respectively, to detect homologous recombination in two dependent ES clones (#1 and #2). Germline (GL) and targeted allele-derived bands are indicated by arrows (left panel), and the recombination-specific PCR product is indicated by an arrowhead (right panel). (C) Western blotting of A20 expression in A20*^flox/flox^ MxCre* ^+^ mice. Proteins extracted from the spleens of LPS-stimulated A20*^flox/flox^ MxCre* ^−^ and A20*^flox/flox^ MxCre* ^+^ mice were blotted and probed with anti-A20- (upper panel) or anti-β-actin antibodies (lower panel). The position of A20 is indicated by an arrow.
(PDF)

Figure S2 **Proliferation of mature myeloid cells in the spleen of** ***A20Mx*** **and** ***A20Mx*** **BMT mice.** Higher magnification of HE-stained sections of the spleen of *A20Mx* and *A20Mx* BMT mice. Mature myeloid cells with segmented or multi-lobulated nuclei are indicated by arrowheads.
(PDF)

Figure S3 **Apoptosis of B cells.** Representative results of double staining with an anti-B cell antibody and TUNEL in *control* and *A20Mx* spleens (three weeks old). Blue and brown staining patterns show B and apoptotic cells, respectively. The boxed areas in the upper panels are magnified in the lower panels.
(PDF)

Figure S4 **Analysis of** ***control*** **and** ***A20ERT2*** **mice.** (A) HE-stained *A20ERT2* tissues that show severe apoptosis. Microemboli and necrotic areas in the liver are indicated by arrowheads and an arrow, respectively. § below standard range and out of invertable range. (B) Serum concentrations of pro-inflammatory cytokines. *p<0.05 and **p<0.01 (Student’s *t*-test). § below standard range and out of invertable range.
(PDF)

Figure S5 **Colony formation assay.** (A) The colony numbers generated in the presence of SCF+IL3+Epo, and those generated with SCF+IL7 are shown. No significant difference was observed between *control* and *A20Mx* mice. (B) Representative images of colonies. Colonies derived from both types of mice are similar in size and shape.
(PDF)

Table S1 **Antibodies.** All the antibodies used in this study are listed.
(PDF)

Table S2 **Characteristics of** ***A20Mx*** **mice.** Hematopoietic parameters of moribund *A20Mx* mice are described.
(PDF)

Text S1 **Construction of a targeting vector and generation of** ***A20*** **knockout mice.** Detailed procedure of Construction of a targeting vector and generation of *A20* knockout mice is described.
(PDF)
