# Supplementary figures and images for: Acquired Deficiency of A20 Results in Rapid Apoptosis, Systemic Inflammation, and Abnormal Hematopoietic Stem Cell Function
Source: PLoS One. 2014 Jan 31;9(1):e87425. doi: 10.1371/journal.pone.0087425 (PMC3909109; doi:10.1371/journal.pone.0087425)

**(A)**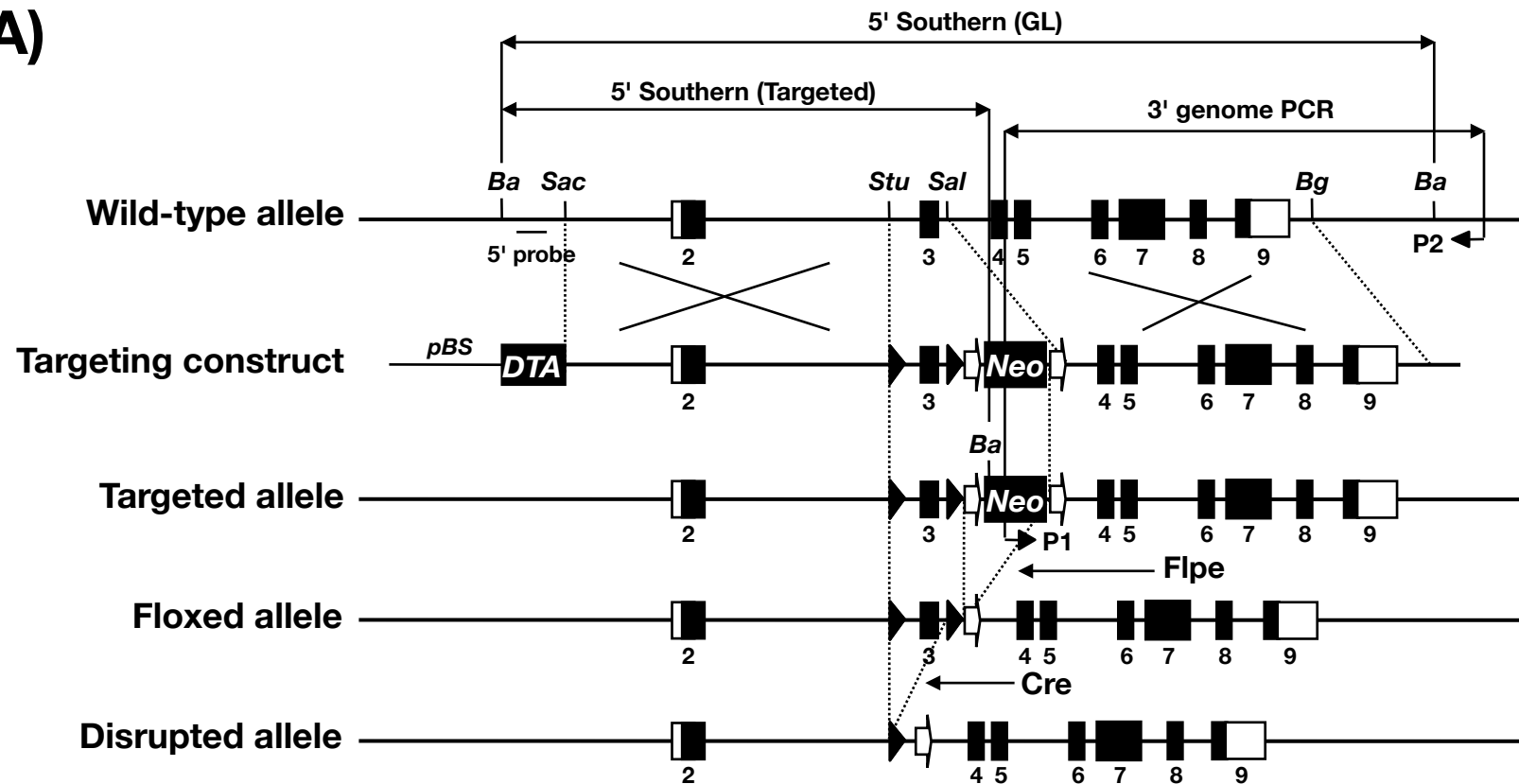**(B)**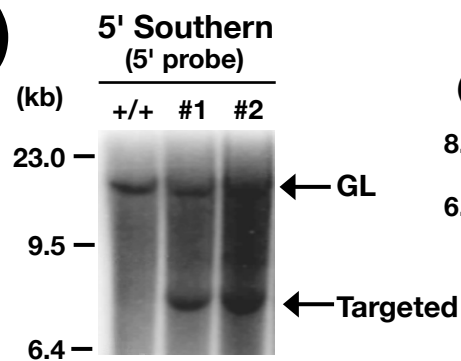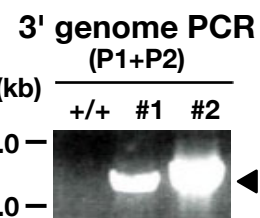**(C)**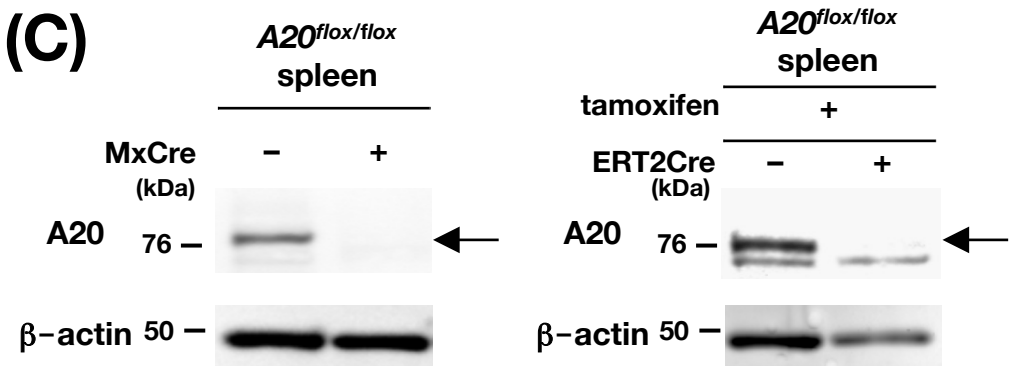Figure S1. Nagamachi A., *et al.*

Supplement: Figure S1 — Generation of A20 conditional knockout mice. (A) Targeting strategy. Exon 3 of mouse A20 was floxed and the Frt-flanked Neo-resistance gene was removed using Flp recombinase. The positions of a 5′ probe for Southern blotting and P1 and P2 primers for 3′ genomic PCR analysis are shown. Restriction sites: Ba, BamHI; Sac, SacI; Stu, StuI; Sal, SalI; Bg, BglII. (B) Southern blotting and genomic PCR using a 5′ probe and 3′ primers, respectively, to detect homologous recombination in two dependent ES clones (#1 and #2). Germline (GL) and targeted allele-derived bands are indicated by arrows (left panel), and the recombination-specific PCR product is indicated by an arrowhead (right panel). (C) Western blotting of A20 expression in A20flox/flox MxCre + mice. Proteins extracted from the spleens of LPS-stimulated A20flox/flox MxCre − and A20flox/flox MxCre + mice were blotted and probed with anti-A20- (upper panel) or anti-β-actin antibodies (lower panel). The position of A20 is indicated by an arrow. (PDF) [file pone.0087425.s001.pdf]

***A20Mx* spleen**

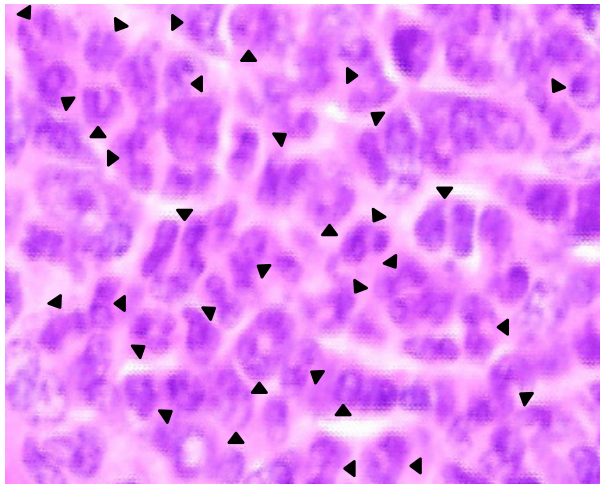

***A20ERT2* spleen**

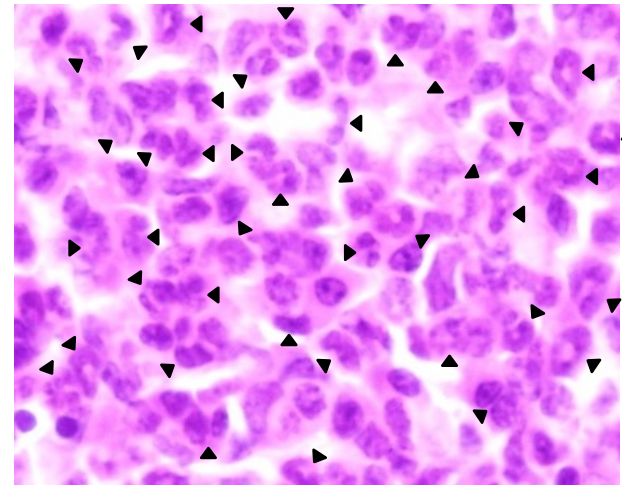

**Figure S2. Nagamachi A., *et al.***

Supplement: Figure S2 — Proliferation of mature myeloid cells in the spleen of A20Mx and A20Mx BMT mice. Higher magnification of HE-stained sections of the spleen of A20Mx and A20Mx BMT mice. Mature myeloid cells with segmented or multi-lobulated nuclei are indicated by arrowheads. (PDF) [file pone.0087425.s002.pdf]

**Control spleen**

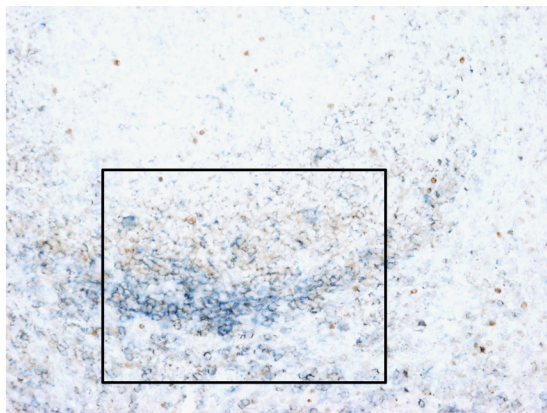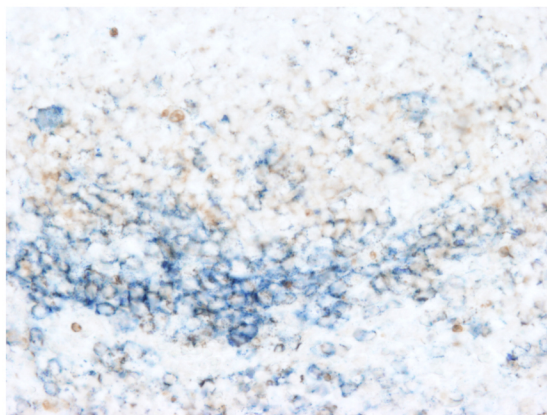

**A20Mx spleen**

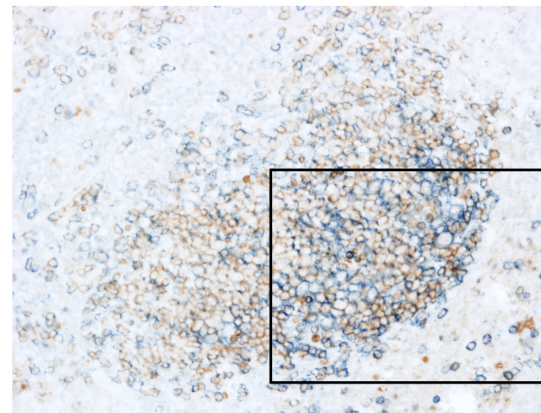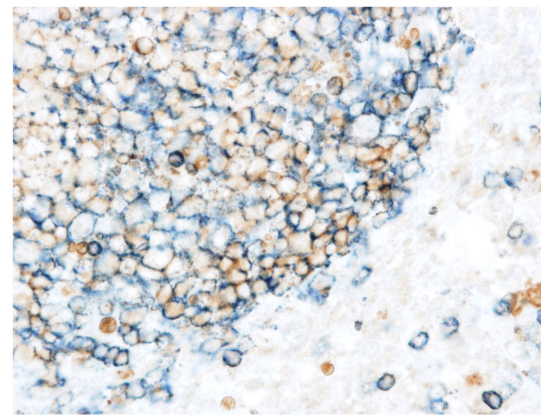

**Figure S3. Nagamachi A., et al.**

Supplement: Figure S3 — Apoptosis of B cells. Representative results of double staining with an anti-B cell antibody and TUNEL in control and A20Mx spleens (three weeks old). Blue and brown staining patterns show B and apoptotic cells, respectively. The boxed areas in the upper panels are magnified in the lower panels. (PDF) [file pone.0087425.s003.pdf]

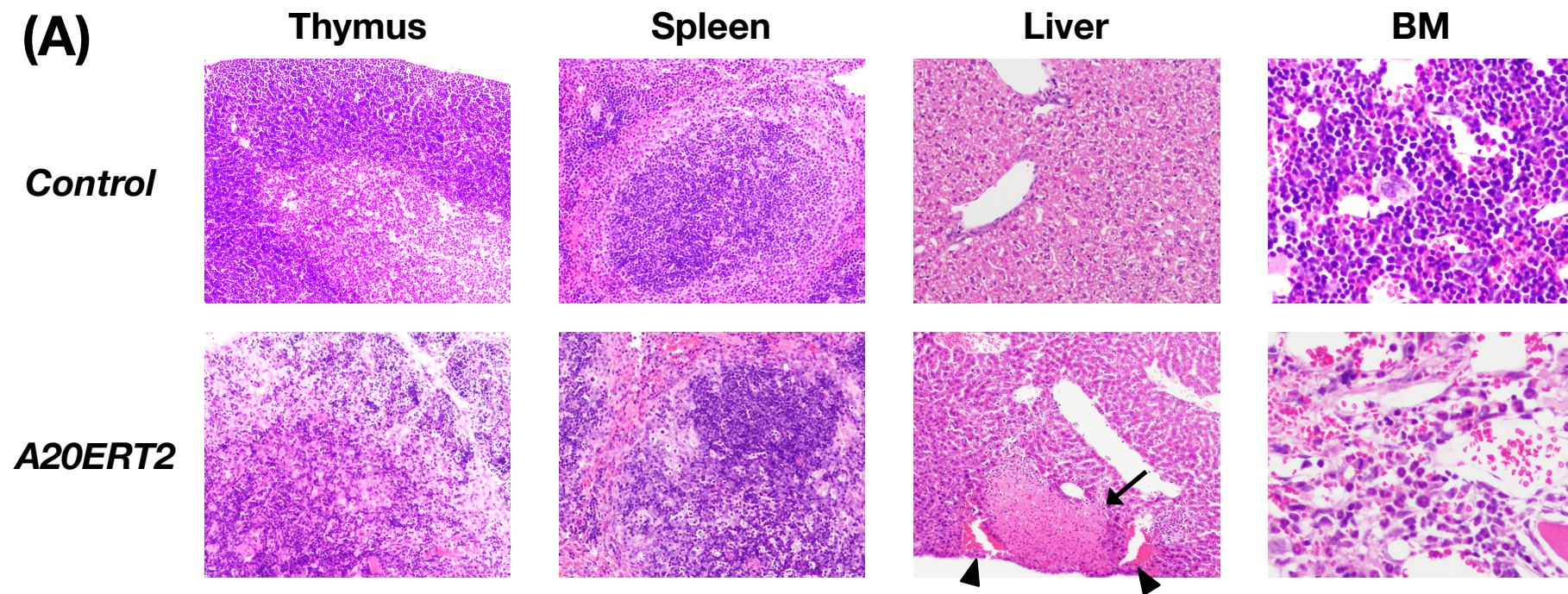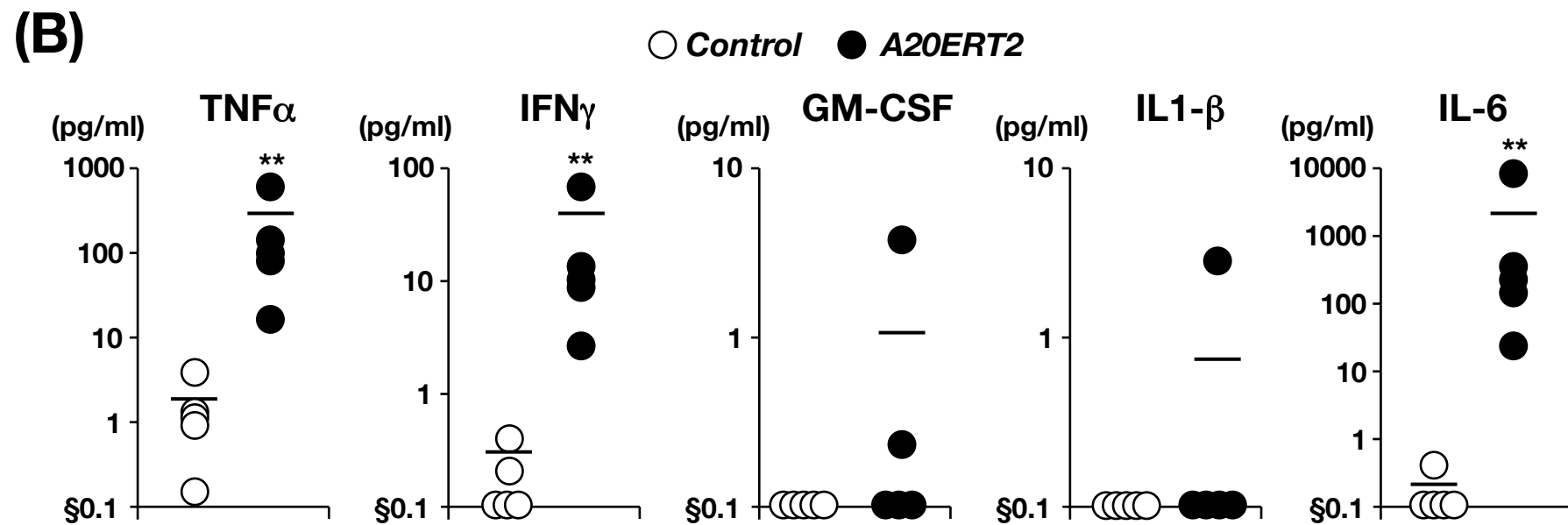

Figure S4. Nagamachi A., *et al.*

Supplement: Figure S4 — Analysis of control and A20ERT2 mice. (A) HE-stained A20ERT2 tissues that show severe apoptosis. Microemboli and necrotic areas in the liver are indicated by arrowheads and an arrow, respectively. § below standard range and out of invertable range. (B) Serum concentrations of pro-inflammatory cytokines. *p<0.05 and **p<0.01 (Student’s t-test). § below standard range and out of invertable range. (PDF) [file pone.0087425.s004.pdf]

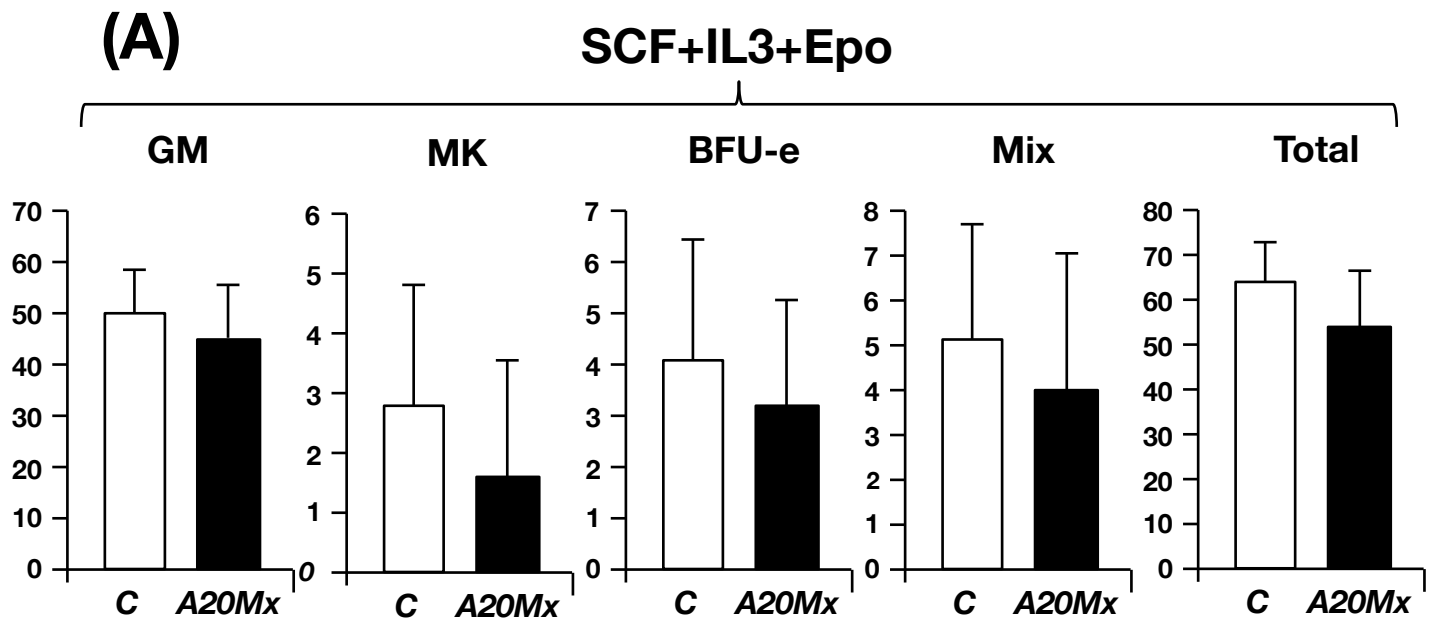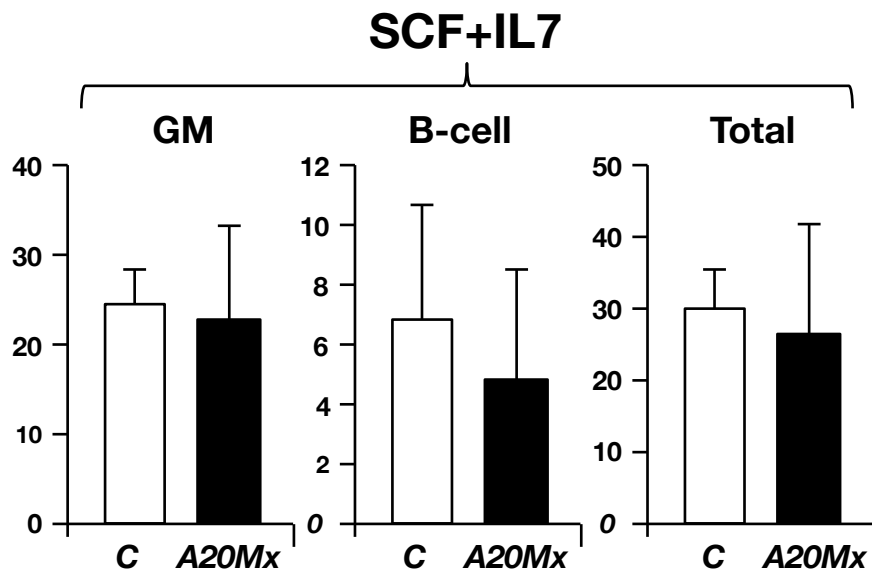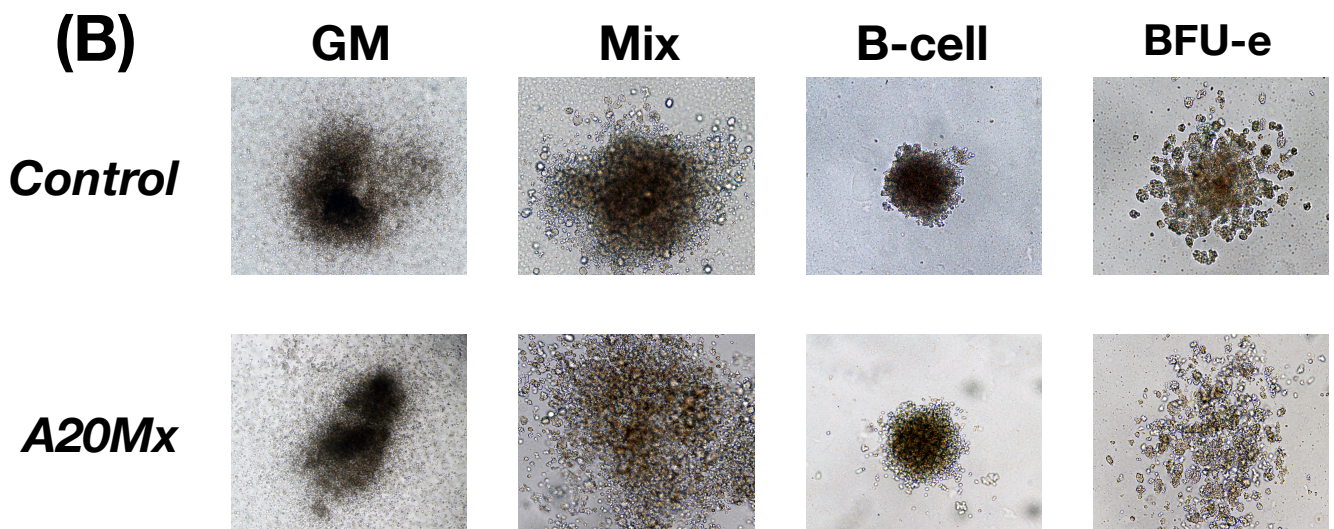

Figure S5. Nagamachi A., et al.

Supplement: Figure S5 — Colony formation assay. (A) The colony numbers generated in the presence of SCF+IL3+Epo, and those generated with SCF+IL7 are shown. No significant difference was observed between control and A20Mx mice. (B) Representative images of colonies. Colonies derived from both types of mice are similar in size and shape. (PDF) [file pone.0087425.s005.pdf]
