# Supplementary material for: Acquired Deficiency of A20 Results in Rapid Apoptosis, Systemic Inflammation, and Abnormal Hematopoietic Stem Cell Function
Source: PLoS One. 2014 Jan 31;9(1):e87425. doi: 10.1371/journal.pone.0087425 (PMC3909109; doi:10.1371/journal.pone.0087425)
Supplement: Table S1 — Antibodies. All the antibodies used in this study are listed. (PDF) [file pone.0087425.s006.pdf]

Table S1

Antibodies for Western blot and immunofluorescent analyses

Anti-A20 antibody for Western blot

A20/TNFAIP3 (D13H3) rabbit mAb (Cell signaling Technology #5630, ×500)

Anti-NF-κB antibody for immunofluorescent staining

NF-κB p65 (D14E12) XP rabbit mAb (Cell signaling Technology #8242, ×500)

Antibodies for flow cytometric analysis

anti-CD45R/B220-PE-Cy7 (RA3-6B2) (BioLegend #103222)
anti-CD90.2 (Thy1.2)-FITC (30-H12) (BD pharmingen™ #553013)
anti-CD11b-FITC (M1/70) (BD pharmingen™ #553310)
anti-Ly6G/Ly6C (Gr-1)-PE-Cy7 (RB6-8C5) (BD pharmingen™ #108416)
anti-Ter-119-APC (BD pharmingen™ #557909)
anti-CD45.1-APC (A20) (BD pharmingen™ #558701)
anti-CD45.2-PE (104) (eBioscience #12-0454-82)
anti-CD117 (c-Kit)-APC-H7 (2B8) (BD pharmingen™ #560185)
anti-Ly-6A/E (Sca-1) (BioLegend #122514)
anti-CD135 (A2F10.1) (BD pharmingen™ #553842)
anti-CD150 (mShad159) (eBioscience #12-1502-80)
anti-CD34-FITC (RAM34) (BD pharmingen™ #560238)
anti-CD4-Biotin (RM4-5) (BD pharmingen™ #553044)
anti-CD41(eBioMWReg30) (eBioscience #11-0411-81)
anti-CD48 (HM48-1) (eBioscience #11-0481-81)
anti-CD8a-Biotin (53-6.7) (BD pharmingen™ #553029)
PerCP-Cy™5.5 Streptavidin (BD pharmingen™ #551319)
Biotin Mouse Lineage Panel (BD pharmingen™ #559971)
anti-CD32/CD16 (2.4G2) (to block Fc receptors) (BD pharmingen™ #553142)

Table S1. Nagamachi A. *et al.*
