# Supplementary material for: Acquired Deficiency of A20 Results in Rapid Apoptosis, Systemic Inflammation, and Abnormal Hematopoietic Stem Cell Function
Source: PLoS One. 2014 Jan 31;9(1):e87425. doi: 10.1371/journal.pone.0087425 (PMC3909109; doi:10.1371/journal.pone.0087425)
Supplement: Table S2 — Characteristics of A20Mx mice. Hematopoietic parameters of moribund A20Mx mice are described. (PDF) [file pone.0087425.s007.pdf]

# Table S2. Characteristics of *A20Mx* mice

| Mouse no. | Age at disease (day) | PB parameters | | | Enlarged organs | FACS parameters |
|---|---|---|---|---|---|---|
| | | WBC ($\times 10^3/\mu l$) | Hb (g/dl) | Plt ($\times 10^4/\mu l$) | | |
| No.1 | 67 | 24.5 | 3.9 | >100 | Spl, LN | $Gr1^+/Mac1^+$ 32.0%, $B220^+$ 14% |
| No.2 | 95 | 14.6 | 5.4 | 59.1 | Spl, LN | $Gr1^+/Mac1^+$ 59.0%, $B220^+$ 8.6% |
| No.3 | 123 | 9.2 | 5.0 | >100 | Spl, LN | $Gr1^+/Mac1^+$ 49.0%, $B220^+$ 17% |
| No.4 | 169 | 7.0 | 12.4 | 30.0 | Spl, Liv, LN | $Gr1^+/Mac1^+$ 71.6%, $B220^+$ 8.2% |
| No.5 | 170 | 9.9 | 3.0 | >100 | Spl, Liv, LN | $Gr1^+/Mac1^+$ 72.9%, $B220^+$ 4.8% |
| No.6 | 176 | 6.2 | 5.8 | >100 | Spl, Liv | $Gr1^+/Mac1^+$ 60.7%, $B220^+$ 5.8% |
| No.7 | 177 | 12.5 | 8.0 | 69.1 | Spl, Liv, LN | $Gr1^+/Mac1^+$ 71.2%, $B220^+$ 4.6% |
| No.8 | 200 | 5.4 | 8.4 | 98.0 | Spl, Liv | $Gr1^+/Mac1^+$ 53.0%, $B220^+$5.6% |
