# Supplementary material for: Acquired Deficiency of A20 Results in Rapid Apoptosis, Systemic Inflammation, and Abnormal Hematopoietic Stem Cell Function
Source: PLoS One. 2014 Jan 31;9(1):e87425. doi: 10.1371/journal.pone.0087425 (PMC3909109; doi:10.1371/journal.pone.0087425)
Supplement: Text S1 — Construction of a targeting vector and generation of A20 knockout mice. Detailed procedure of Construction of a targeting vector and generation of A20 knockout mice is described. (PDF) [file pone.0087425.s008.pdf]

Text S1

Construction of a targeting vector and generation of *A20* knockout mice

A BAC clone containing mouse *A20* was purchased from BACPAC Resource Center of Children's Hospital Oakland Research Institute (Oakland, CA). *Sac*I–*Stu*I and *Sal*I–*Bgl*II fragments of *A20* were used as the 5′ and 3′ arms of the targeting vector, respectively (Fig. S1A). A *floxed Stu*I-*Sal*I fragment containing exon 3 was inserted between the two arms together with a *flip-recombinase target* (*Frt*)-flanked *neomycin resistance* (*Neo*) gene. A *diphtheria toxin-A* (*DTA*) gene was attached to the 5′ end for negative selection. KY1.1 ES cells (provided by Dr. Takeda) were electroporated with the targeting vector and subjected to G418 selection as described (1). Homologous recombination within ES clones was identified by blotting *Bam*HI-digested genomic DNA with a *Bam*HI–*Sac*I 5′ probe and by 3′ genomic PCR (Fig. S1B). Correctly targeted ES clones were injected into C57BL/6 mouse blastocysts and chimeric males were mated with C57BL/6 females to transmit the targeted allele. *Frt*-flanked *Neo* was removed by crossing the heterozygotes with *CAG-Flpe* transgenic mice (RBRC01834, provided by RIKEN BRC), which generated a *floxed* allele. $A20^{+/flox}$ mice were crossed with *Mx1–Cre* transgenic ($MxCre^{+}$) mice (2) or *ERT2–Cre* ($ERT2Cre^{+}$) mice. The resultant $A20^{+/flox}$ $MxCre^{+}$ mice and $A20^{+/flox}$ $ERT2Cre^{+}$ mice were crossed to generate $A20^{flox/flox}$ $MxCre^{+}$ mice and $A20^{flox/flox}$ $ERT2Cre^{+}$ mice, respectively. $A20^{flox/flox}$ $MxCre^{-}$ and $A20^{flox/flox}$ $ERT2Cre^{-}$ mice were used as controls.

1. Honda H, Oda H, Nakamoto T, Honda Z, Sakai R, Suzuki T, et al. (1998) Cardiovascular anomaly, impaired actin bundling and resistance to Src-induced transformation in mice lacking p130Cas. Nat Genet. 19; 361-365
2. Kuhn R, Schwenk F, Aguet M, Rajewsky K (1995) Inducible gene targeting in mice. Science 269: 1427-1429.
